# Casting your network wide: a plea to scale-up phenological research

**DOI:** 10.1098/rsbl.2016.0181

**Published:** 2016-06

**Authors:** Stephen J. Thackeray

**Affiliations:** Lake Ecosystems Group, Centre for Ecology and Hydrology, Lancaster Environment Centre, Library Avenue, Bailrigg, Lancaster LA1 4AP, UK

**Keywords:** food web, predator–prey, spatial heterogeneity, synchrony, trophic cascade

## Abstract

Accumulating scientific evidence has demonstrated widespread shifts in the biological seasons. These shifts may modify seasonal interspecific interactions, with consequent impacts upon reproductive success and survival. However, current understanding of these impacts is based upon a limited number of studies that adopt a simplified ‘bottom-up’ food-chain paradigm, at a local scale. I argue that there is much insight to be gained by widening the scope of phenological studies to incorporate food-web interactions and landscape-scale processes across a diversity of ecosystem types, with the ultimate goal of developing a generic understanding of the systems most vulnerable to synchrony effects in the future. I propose that co-location of predator and prey phenological monitoring at sentinel sites, acting as research platforms for detailed food-web studies, experimentation and match-up with earth observation data, would be an important first step in this endeavour.

## 1. Introduction

Shifts in the biological seasons indicate that climate change is already impacting ecosystems [1]. These phenological changes are near ubiquitous: manifest in the seasonal activities of many taxa [2–4]. However, observed among-species differences in rates of changing seasonality have led to concerns that historically synchronized seasonal species interactions will become de-synchronized [5]. Effects on trophic interactions are widely discussed (*sensu* the ‘match–mismatch’ hypothesis [6]), whereby consumer populations are predicted to decline when the seasonal timing of their peak energy demand changes at a different rate from the seasonal timing of prey/resource peaks.

In addition to the marine systems, in which the concept of match–mismatch originated, there are now further field demonstrations of the potential importance of synchrony for consumer reproductive success in seasonal environments. Breeding success in insectivorous passerines is linked to the relative seasonal timing of chick provisioning and peaks in caterpillar food resources [7], whereas survival of caribou calves correlates with the relative timing of seasonal vegetative growth and birth date [8]. In the freshwater environment, perch recruitment is partially dependent upon the seasonality of spawning compared with seasonal food resources [9].

We need to understand and quantify the impacts of changing synchrony, in order to improve our ability to predict future ecological impacts of phenological shifts. Existing studies show potential impacts of match–mismatch, but we lack the understanding needed to make general inferences regarding the species and ecosystem types most vulnerable to these effects. I argue that, to move beyond the state-of-the-art, we must make the ambitious conceptual leap to view match–mismatch within the context of complex networks of reciprocal ecological interactions, in a spatially heterogeneous environment.

## 2. Food chains versus food webs

Current studies on match–mismatch largely adopt a simplified food-chain paradigm, comparing synchrony between single consumer populations and either aggregate prey availability or individual prey species [7,9,10]. This simplification, justified for analytical tractability, is conceptually appropriate for interactions involving trophic specialists. However, many consumers are trophic generalists [11,12], switching between multiple prey types. For generalists, it is plausible that reduced synchrony with one prey species will result in intensified predation upon other species. Therefore, we would expect that phenological change does not ‘break apart’ food chains, but in fact modifies relative interaction strengths within food webs. Despite the possibility for phenological change to bring about novel species interactions, research focus has been on the weakening of existing interactions. A priority is to understand whether shifts in prey use could afford a degree of resilience to generalist predators.

Although generalists may offset mismatching by prey-switching, alternative food resources are unlikely to be totally equivalent in nutritional quality and energetic value. Furthermore, prey will differ in handling time, ease of capture and with respect to the foraging time/energy that a predator has to invest in order to locate them. Therefore, for generalist predators within food webs, phenological change will likely exert as much influence through variations in prey ‘quality’ as through changes in prey quantity.

**Figure 1.**
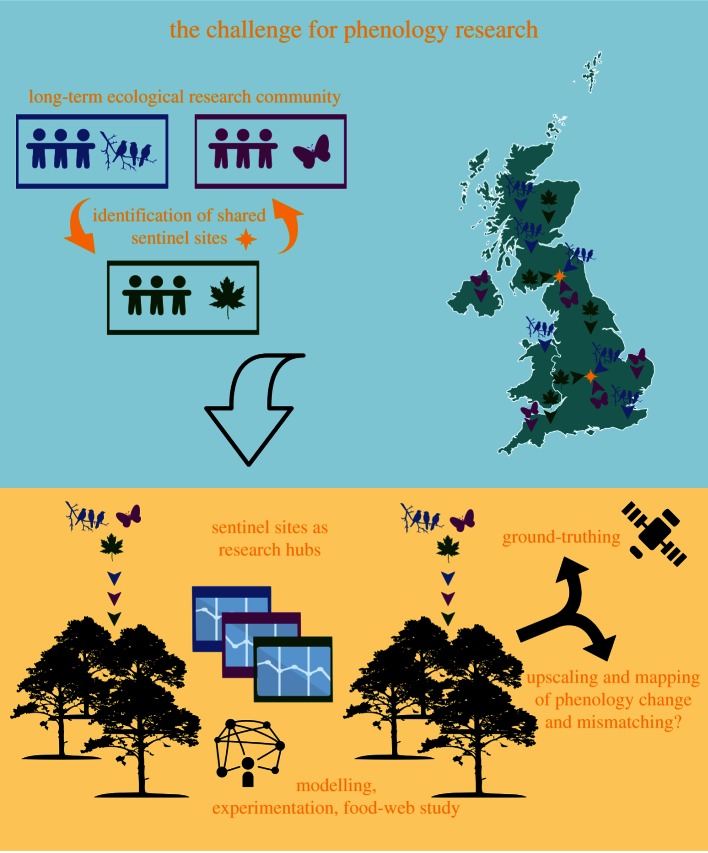
A challenge for phenological research is to align monitoring schemes around shared sentinel sites, representing ecosystem types and acting as hubs for fundamental research into causes and consequences of mismatching at the ecosystem scale.

Our capacity to monitor food-web effects of phenological change in the field is currently limited by a relative scarcity of co-located phenological monitoring for generalist predators and multiple prey taxa. Different food-web components (e.g. plants, insects and birds) are frequently monitored by different organizations or initiatives, varying in their underlying research motivations. To develop a more detailed mechanistic understanding of the relationships between phenological change and ‘bottom-up’ effects upon consumers, we could align some of this biological monitoring around sentinel sites where phenological data are collected for multiple food-web components (figure 1). This would yield data on the relative seasonal timing of predators and multiple prey, but to understand ecological consequences would require the further step of integrating phenology and food-web research. I propose that the sentinel sites would operate as research platforms for detailed food-web studies that would compare the dietary composition of, and energy sources used by, ‘early’ and ‘late’ consumer individuals using techniques such as stable isotopes [13] and traditional/meta-barcoding approaches to gut contents analysis [14], coupled with determinations of the ‘quality’ of different prey. This would allow the construction of networks of species interactions and energy flows and the comparison of food-web architecture (e.g. food-chain length, mean trophic position and connectance) around consumers with differing phenological traits. Monitoring organizations will need to continue to gather data at long-running sites in order to address their primary research objectives, but sentinel sites would be embedded within these existing monitoring networks. For example, sentinel sites could be established within existing networks such as the National Ecological Observatory Network (http://www.neonscience.org/) and Track a Tree (http://trackatree.bio.ed.ac.uk/). Ideally, the series of sentinel sites would represent different ecosystem types within each major environment. For example, different sentinel sites would represent temperate deciduous forests and semi-natural grasslands in the terrestrial environment, and lakes and rivers in the freshwater environment, providing data that will fuel a more generic understanding of the food-web implications of phenological change.

## 3. Bottom-up versus top-down

The match–mismatch concept was, at its inception, ‘bottom-up’: focusing upon the consequences of synchrony with food resources for predator recruitment/breeding success [6]. However, phenologically altered interaction strengths between predators and multiple prey may impact ‘top-down’ upon prey dynamics, propagating throughout food-webs and ecological networks [15,16]. We may therefore view match–mismatch as a key mechanism behind the more generic trophic cascade phenomenon [17].

Trophic cascade theory predicts that changes in predator biomass will impact ‘top-down’ upon dynamics at lower trophic levels [17,18]. However, theoretical models suggest that alterations to the seasonal timing of predator emergence or energy demand will also mediate the intensity of ‘top-down’ control [15]. These predicted top-down effects remain at the theoretical stage and are central to understanding ecosystem-scale effects of altered synchrony. Although widely reported, the strength of the traditional abundance-mediated trophic cascade is highly variable among systems and species groups [19]. It is likely that the same will be true of phenologically mediated cascades. It is important that we confront theoretical expectations [15] with scenarios from models that simulate specific ecosystems with a greater degree of realism and with community-level observational data from sentinel sites. Ecosystem experiments could also shed light onto these potential cascading effects. For example, replicated mesocosm experiments could be used to track emerging differences in community succession in response to an experimental treatment in which predator phenology is directly manipulated. This approach would be especially powerful if the mesocosms mimicked communities monitored at the proposed sentinel sites, adding process understanding to observations of change. Together, these approaches would allow us to identify the species interactions and ecosystems that do or do not conform to theoretical expectations. We must develop appropriate ecosystem models where they are lacking, undertake experimental manipulations of phenology and community-level monitoring to address this knowledge gap.

## 4. The spatial dimension

Much contemporary research into trophic mismatching has focused upon interactions at single locations or in habitat patches that are implicitly assumed to be spatially homogeneous [20,21]. This is necessitated by constraints upon resources available to support long-term monitoring activities. However, phenological traits are spatially heterogeneous at a range of scales [22–25]. This heterogeneity is not simply a ‘nuisance’ to be accounted for when designing representative sampling programmes. It is ecologically relevant structure that has implications for the intensity of bottom-up and top-down food-web effects of phenological change and for emergent demographic properties.

Spatial processes influence phenological synchrony and its implications through day-to-day foraging movements, meta-population dynamics and migration. The latter process affects when consumers reach their breeding grounds, the former processes affect match–mismatch effects once there. The foraging behaviour of individual consumers affects the spatial scale over which their phenologically patchy environment is experienced and effectively ‘sampled’. Through short-term modification of foraging movements, consumers may escape local mismatching by focusing on patches with more optimal resource seasonality and not patches in which there is poor synchrony with food resources. Indeed, landscape-scale vegetation phenology is believed important to invertebrate consumers in some Arctic systems [24].

We can also view distinct populations of consumers in a phenologically patchy landscape from a meta-population perspective. Local populations of consumers may fine-tune reproductive timing to prey phenology in particular locations via plasticity [25] or local adaptation [22]. If the extent of plasticity or adaptation varies spatially, then the extent of match–mismatch might also vary spatially, i.e. synchrony with prey/resources in some places but not others. Such heterogeneity may allow a consumer species to persist despite severe, local, desynchronization in some habitat patches.

The abovementioned mechanisms might confer consumers with some resilience to localized phenological mismatching, but are challenging to observe. Replicated sentinel sites within the same ecosystem type, but at different locations, would allow assessment of spatial coherence in phenological food-web effects, to understand whether localized mismatching is compensated for in other habitat patches. It is likely that many replicate sentinel sites would be needed for each ecosystem type if the ultimate goal is to understand the drivers of observed differences. Upscaling beyond these sites to a broad-scale assessment of match–mismatch might be facilitated by the increasing availability of earth observation data to ecologists. Satellite data can quantify spatial variation in vegetation (including planktonic primary producer) phenology at different scales, and in different habitat types, but yield no information on consumer organisms. The challenge is to ‘ground-truth’ satellite data against *in situ* biological observations of primary producers and then, by also ‘matching up’ with gridded climate and *in situ* consumer data, to investigate whether empirical models can be constructed to make tentative predictions and maps of spatio-temporal changes in consumer phenology based upon seasonal change in vegetation and climate over broad spatial scales (figure 1). These changes could then be related to *in situ* observations of fitness consequences for consumers at sentinel sites [25].

## 5. Conclusion

Phenological change has the potential to modify ecological processes and interactions. However, it is possible that processes operating at the scale of ecological networks and landscapes may have a profound influence on the magnitude of these effects. The time has come to work towards a more generic predictive capability that allows us to quantify impacts at different biological scales, assess what proportion of species will be affected, and determine which types of species and system are likely to be least resilient to change. By aligning phenological monitoring for interacting species around a series of sentinel sites representing different ecosystem types, we would create research hubs allowing collaboration across disciplinary boundaries, and integration of demographic and ecological data and expertise (figure 1). This approach would be an important first step in scaling-up phenological research to food webs and landscapes. Conservation biology would greatly benefit from the scientific understanding that we would gain from this endeavour, which would help resolve how phenology intersects with species interactions and landscape connectivity to influence population trajectories.

## References

[1] IPCC. 2014 Climate change 2014: impacts, adaptation, and vulnerability. Part A: global and sectoral aspects. In Contribution of working group II to the fifth assessment report of the intergovernmental panel on climate change (eds Field CB, Barros VR, Dokken DJ), 1132 pp. Cambridge, UK: Cambridge University Press.

[2] Parmesan C, Yohe G 2003 A globally coherent fingerprint of climate change impacts across natural systems. Nature 421, 37–42. (10.1038/nature01286) 12511946

[3] Root TL, Price JT, Hall KR, Schneider SH, Rosenzweig C, Pounds JA 2003 Fingerprints of global warming on wild animals and plants. Nature 421, 57–60. (10.1038/nature01333) 12511952

[4] Thackeray SJ et al. 2010 Trophic level asynchrony in rates of phenological change for marine, freshwater and terrestrial environments. Glob. Change Biol. 16, 3304–3313. (10.1111/j.1365-2486.2010.02165.x)

[5] Visser ME, Both C 2005 Shifts in phenology due to global climate change: the need for a yardstick. Proc. R. Soc. B 272, 2561–2569. (10.1098/rspb.2005.3356) PMC1559974 16321776

[6] Cushing DH 1990 Plankton production and year class strength in fish populations—an update of the match/mismatch hypothesis. Adv. Mar. Biol. 26, 249–293. (10.1016/S0065-2881(08)60202-3)

[7] Both C, van Asch M, Bijlsma RG, van den Burg AB, Visser ME 2009 Climate change and unequal phenological changes across four trophic levels: constraints or adaptations? J. Anim. Ecol. 78, 73–83. (10.1111/j.1365-2656.2008.01458.x) 18771506

[8] Post E, Forchhammer MC 2008 Climate change reduces reproductive success of an Arctic herbivore through trophic mismatch. Phil. Trans. R. Soc. B 363, 2367–2373. (10.1098/rstb.2007.2207) PMC2606787 18006410

[9] Ohlberger J, Thackeray SJ, Winfield IJ, Maberly SC, Vøllestad LA 2014 When phenology matters: age–size truncation alters population response to trophic mismatch. Proc. R. Soc. B 281, 20140938 (10.1098/rspb.2014.0938) PMC4173671 25165767

[10] Winder M, Schindler DE 2004 Climate change uncouples trophic interactions in an aquatic ecosystem. Ecology 85, 2100–2106. (10.1890/04-0151)

[11] Miller-Rushing AJ, Høye TT, Inouye DW, Post E 2010 The effects of phenological mismatches on demography. Phil. Trans. R. Soc. B 365, 3177–3186. (10.1098/rstb.2010.0148) 20819811 PMC2981949

[12] Polis GA, Strong DR 1996 Food web complexity and community dynamics. Am. Nat. 147, 813 (10.1086/285880)

[13] Layman CA et al. 2012 Applying stable isotopes to examine food-web structure: an overview of analytical tools. Biol. Rev. 87, 545–562. (10.1111/j.1469-185X.2011.00208.x) 22051097

[14] Razgour O, Clare EL, Zeale MRK, Hanmer J, Schnell IB, Rasmussen M, Gilbert TP, Jones G 2011 High-throughput sequencing offers insight into mechanisms of resource partitioning in cryptic bat species. Ecol. Evol. 1, 556–570. (10.1002/ece3.49) 22393522 PMC3287336

[15] Nakazawa T, Doi H 2012 A perspective on match/mismatch of phenology in community contexts. Oikos 121, 489–495. (10.1111/j.1600-0706.2011.20171.x)

[16] Christoffersen K, Riemann B, Klysner A, SØndergaard M 1993 Potential role of fish predation and natural populations of zooplankton in structuring a plankton community in eutrophic lake water. Limnol. Oceanogr. 38, 561–573. (10.4319/lo.1993.38.3.0561)

[17] Carpenter SR, Kitchell JF, Hodgson JR 1985 Cascading trophic interactions and lake productivity. Bioscience 35, 634–639. (10.2307/1309989)

[18] Brett MT, Goldman CR 1996 A meta-analysis of the freshwater trophic cascade. Proc. Natl Acad. Sci. USA 93, 7723–7726. (10.1073/pnas.93.15.7723) 11607694 PMC38814

[19] Borer ET, Seabloom EW, Shurin JB, Anderson KE, Blanchette CA, Broitman B, Cooper SD, Halpern BS 2005 What determines the strength of a trophic cascade? Ecology 86, 528–537. (10.1890/03-0816)

[20] Thackeray SJ, Henrys PA, Feuchtmayr H, Jones ID, Maberly SC, Winfield IJ 2013 Food web de-synchronization in England's largest lake: an assessment based on multiple phenological metrics. Glob. Change Biol. 19, 3568–3580. (10.1111/gcb.12326) 23868351

[21] Reed TE, Grøtan V, Jenouvrier S, Sæther B-E, Visser ME 2013 Population growth in a wild bird is buffered against phenological mismatch. Science 340, 488–491. (10.1126/science.1232870) 23620055

[22] Phillimore AB, Hadfield JD, Jones OR, Smithers RJ 2010 Differences in spawning date between populations of common frog reveal local adaptation. Proc. Natl Acad. Sci. USA 107, 8292–8297. (10.1073/pnas.0913792107) 20404185 PMC2889515

[23] Romare P, Schindler DE, Scheuerell MD, Scheuerell JM, Litt AH, Shepherd JH 2005 Variation in spatial and temporal gradients in zooplankton spring development: the effect of climatic factors. Freshw. Biol. 50, 1007–1021. (10.1111/j.1365-2427.2005.01386.x)

[24] Høye TT, Post E, Schmidt NM, Trøjelsgaard K, Forchhammer MC 2013 Shorter flowering seasons and declining abundance of flower visitors in a warmer Arctic. Nat. Clim. Change 3, 759–763. (10.1038/nclimate1909)

[25] Cole EF, Long PR, Zelazowski P, Szulkin M, Sheldon BC 2015 Predicting bird phenology from space: satellite-derived vegetation green-up signal uncovers spatial variation in phenological synchrony between birds and their environment. Ecol. Evol. 5, 5057–5074. (10.1002/ece3.1745) 26640682 PMC4662320

